# A conserved neuronal DAF-16/FoxO plays an important role in conveying pheromone signals to elicit repulsion behavior in *Caenorhabditis elegans*

**DOI:** 10.1038/s41598-017-07313-6

**Published:** 2017-08-03

**Authors:** Donha Park, Jeong-Hoon Hahm, Saeram Park, Go Ha, Gyeong-Eon Chang, Haelim Jeong, Heekyeong Kim, Sunhee Kim, Eunji Cheong, Young-Ki Paik

**Affiliations:** 1 Department of Biochemistry, Yonsei University, Seoul, Korea; 2 Yonsei Proteome Research Center, Yonsei University, Seoul, Korea; 3 Department of Integrated Omics for Biomedical Science, Yonsei University, Seoul, Korea; 4 Department of Biotechnology, and College of Life Science and Biotechnology, Yonsei University, Seoul, Korea; 5 Center for Plant Aging Research, Institute for Basic Science (IBS), Daegu, 42988 Republic of Korea

## Abstract

Animals use pheromones as a conspecific chemical language to respond appropriately to environmental changes. The soil nematode *Caenorhabditis elegans* secretes ascaroside pheromones throughout the lifecycle, which influences entry into dauer phase in early larvae, in addition to sexual attraction and aggregation. In adult hermaphrodites, pheromone sensory signals perceived by worms usually elicit repulsion as an initial behavioral signature. However, the molecular mechanisms underlying neuronal pheromone sensory process from perception to repulsion in adult hermaphrodites remain poorly understood. Here, we show that pheromone signals perceived by GPA-3 is conveyed through glutamatergic neurotransmission in which neuronal DAF-16/FoxO plays an important modulatory role by controlling glutaminase gene expression. We further provide evidence that this modulatory role for DAF-16/FoxO seems to be conserved evolutionarily by electro-physiological study in mouse primary hippocampal neurons that are responsible for glutamatergic neurotransmission. These findings provide the basis for understanding the nematode pheromone signaling, which seems crucial for adaptation of adult hermaphrodites to changes in environmental condition for survival.

## Introduction

Pheromones serve as a chemical language through which organisms of the same species communicate in response to environmental changes, including the presence of stress, different sexes and food scarcity. The soil nematode Caenorhabditis elegans, one of the most genetically well-understood metazoans secretes pheromones termed daumones or ascaroside pheromones throughout the lifecycle. For instance, the nematode ascaroside pheromones have been known to signal worms to enter dauer phase, a non-aging state, under unfavorable growth conditions 1–5. In addition, these ascaroside pheromones (pheromones) are involved in diverse biological processes (e.g., sexual attraction, aggregation, and fungal traps) depending on their developmental stage (early larvae vs. adults) and sex (hermaphrodites vs. male) 6–9. Especially, it is well known that these pheromones act as a signal to the nematode that the surrounding environment is an unfavorable condition. Thus, when pheromone signals are recognized, young larvae enter the dauer phase, a non-aging state, for a long-term survival^1–4^. However, when adult hermaphrodites sense the pheromones, they elicit repulsion response as an initial behavioral output^10^. Although this repulsion serves as a signature of pheromone sensory process, molecular mechanism underlying pheromone sensory process after an initial perception remains less characterized.

In search of pheromone signaling perception, there have been a few reports on the putative pheromone receptors that are demonstrated to mediate neuronal responses to ascr#1–3 in ASK neurons^11^ (﻿SRBC64/66) or receptors in ASI neurons that respond to ascr#2^12^ (DAF-37/38). Additionally, Bargmann group reported the identification of *srg-36/37* genes encode G-protein-coupled receptors for ascr#5 (C3)^13^. Despite decades-long research on pheromone function, its perception, the molecular pathway in adult hermaphrodites that starts from an initial pheromone perception to elicit behavioral outputs as a repulsive response is not fully understood. Here, we show that pheromone sensory signals are likely conveyed through glutamatergic neurotransmission in which neuronal DAF-16/FoxO plays an important modulatory role.

## Results and Discussion

### Perception of pheromone sensory signaling by GPA-3 via insulin/IGF-1 pathway

To identify some molecular components involved in perception of pheromones, we screened G-protein subunit genes by assessing the chemotaxis index of *C. elegans* that had been exposed to three major ascaroside pheromones (daumones 1–3 or ascr#1–3) as a measure of perception of pheromones^8^ (Fig. S1a). These three pheromones have been known as most abundant and highly active on pheromone functions among those identified so far^3, 5, 14, 15^. In our experiment, we used both plate-based assays and drop assays in our study. For instance, the plate-based chemotaxis assay^10^ was used to determine the stationary response to the aversive chemicals at the period of certain times (duration), whereas the drop assay was used especially when the rescuing transgenic animals were not stable lines^16^. Transmission rate of the extrachromosomal arrays in transgenic worms are variable between lines. Some siblings from transgenic worms tend to lose transgene. Thus transgenic animals, which only showed *myo-3P::dsRed* marker (proof of carrying transgene) were individually picked and tested for the drop assay. However, these two assays produced essentially the same results against all three pheromones.

When we determined net movement rather than individual real-time movements of worms across 1 h (Fig. S1a), N2 wild type worms showed a dose- and time-dependent repulsion response to all three individual pheromones (Fig. S1b,c), to which late larvae (L4) and young adult worms responding more strongly than early larvae (L1) (Fig. S1d). Notably, all three pheromones elicited a similar pattern and intensity of repulsion responses. Therefore, we used a single pheromone rather than blend of their combination for the plate-based chemotaxis assay in most cases. It appears that they share the common repulsion behavior in response to any of three pheromones. Because the daumone 1 (ascr#1) induced the repulsion of hermaphrodites as well as induction of dauers and fungal traps^3, 9, 14^, we used mostly daumone 1 in chemotaxis screening of G-protein subunit mutant strains and related experiments.

As the initial pheromone sensory process relies on G-proteins (e.g., GPA-2 and 3)^17^, we further sought to define the specific Gα subunit that primarily transfers the initial detection signals of the three major pheromones. Of the 17 Gα subunits examined, only *gpa-3* mutant worms showed defective repulsion responses to all three pheromones (Figs S1e and S2a), suggesting that *gpa-3* is the major G-protein subunit gene that is involved in the perception of these pheromones. Previously, we reported that *gpa-3* negatively controls both insulin/IGF-1 signaling (IIS) and TGF-beta signaling^18^. Thus, to identify the downstream cell signaling pathway involved in GPA-3 signaling, we assessed repulsion responses in worms with mutations in downstream effectors of IIS or TGF-β pathways. Interestingly, *daf-16/FoxO* mutant worms showed reduced repulsion responses to all three pheromones (Fig. S2b), whereas *daf-3/Smad* and *daf-5/SnoSki* mutant worms showed similar repulsion responses as wild-type worms (Fig. S2c). These results suggest that at least part of the IIS pathway, but not the TGF-β pathway, participates in pheromone sensory signaling in hermaphrodites (Fig. S2b,c). It also indicated that DAF-16/FoxO may play an important role in pheromone sensory signaling process.

### Role of neuronal DAF-16/FoxO in glutamatergic neurotransmission of pheromone sensory signals

**Figure 1:**
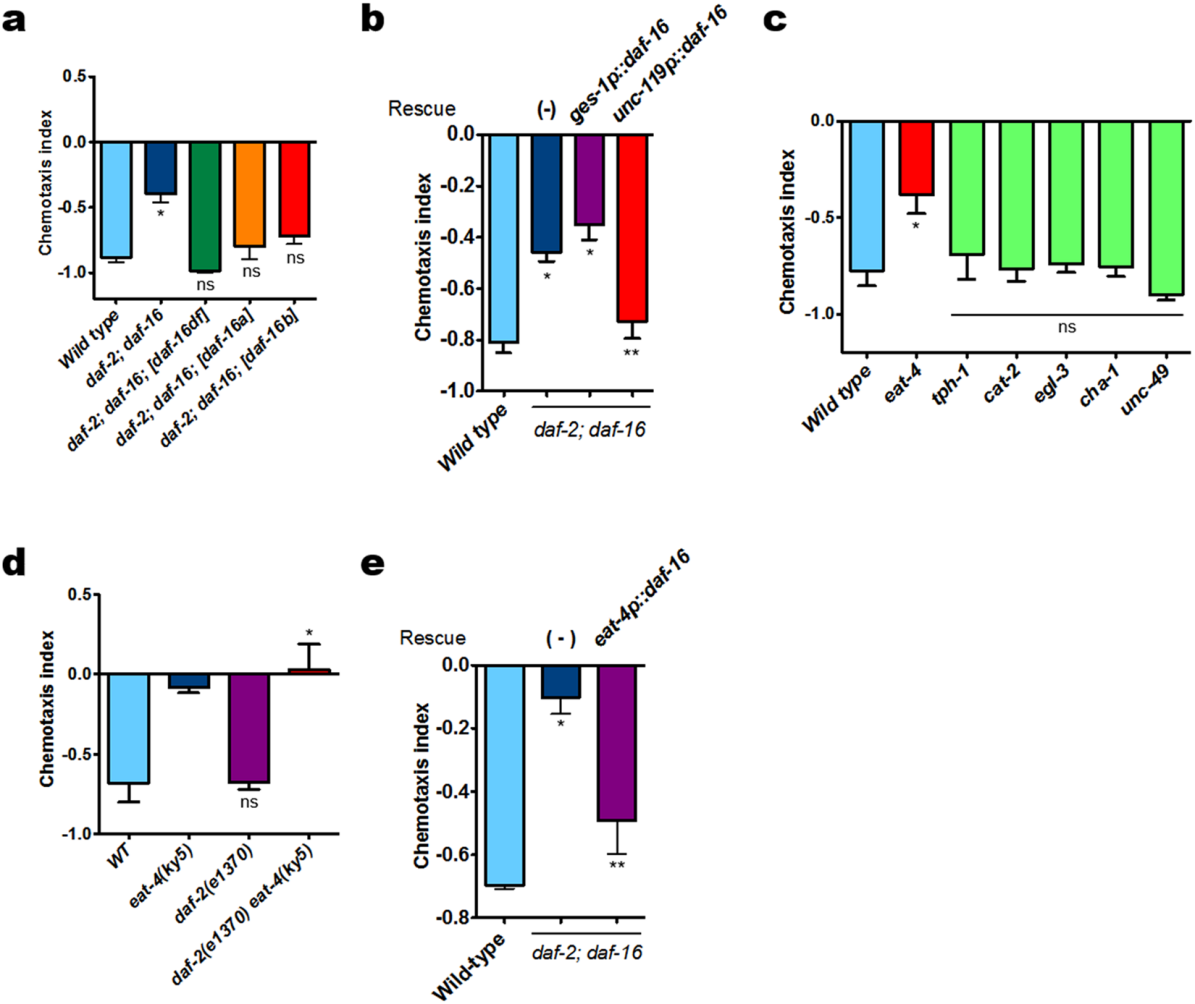
Glutamate signaling mediates pheromone sensory signals to produce repulsion response through the insulin/IGF-1 signaling. (**a**) Rescuing the *daf-2*(*e1370*)*;daf-16/FoxO* (*mgDf50*) phenotype with different *daf-16/FoxO* isoforms (wild type, *n* = 145; *daf-2;daf-16/FoxO*, *n* = 196; *daf-2;daf-16/FoxO*;*DAF-16/FoxOa*, *n* = 146; *daf-2;daf-16/FoxO*;*DAF-16/FoxOdf*, *n* = 218; *daf-2;daf-16;DAF-16/FoxOb*, *n* = 133). (**b**) Tissue-specific rescue of *daf-2;daf-16/FoxO* phenotype (wild type, *n* = 149; *daf-2;daf-16/FoxO, n* = 225; *daf-2;daf-16/FoxO*;*ges-1P::DAF-16/FoxO*, *n* = 150; *daf-2;daf-16/FoxO;unc-119P::DAF-16/FoxO*, *n* = 142). *DAF-16/FoxO* cDNA was expressed under control of intestine- (*ges-1* *P*) or pan-neuronal-specific (*unc-119* *P*) promoters. (**c**) *eat-4*(*ky5*) mutants deficient in glutamate transporter showed defective repulsion responses. (**d**) Genetic epistasis between *daf-2* and *eat-4* mutants (wild type, *n* = 58; *eat-4*, *n* = 47; *daf-2*, *n* = 68*; daf-2 eat-4*, *n* = 62). (e) DAF-16/FoxO in glutamatergic neurons rescued *daf-2;daf-16/FoxO* repulsion responses (wild type, n = 79; *daf-2;daf-16/FoxO*, n = 94; *daf-2;daf-16/FoxO*; *eat-4P::daf-16/FoxO*, n = 80). *DAF-16/FoxO* cDNA was expressed under control of the *eat-4* promoter. *P<0.05, ns: not significant compared to wild type. **P<0.05 compared to *daf-2;daf-16/FoxO*. Significance was determined using two-tailed, unpaired *t*-tests. In these experiments, daumone 1 (1 μ﻿﻿M) was singly used in isolation.

Since DAF-2 normally suppresses nuclear localization of DAF-16/FoxO^19^, we examined whether *daf-16/FoxO* and/or its isoforms participate in the pheromone sensory process. To this end, *daf-2*(*e1370*)*;daf-16/FoxO* (*mgDf50*) double mutants were subjected to rescue experiment by microinjection of those constructs containing each *daf-16/FoxO* isoform^20^. All isoforms examined rescued the repulsion responses of *daf-16/FoxO* mutants, suggesting their common roles in conveying pheromone sensory signals (Fig. 1a). Notably, the rescue effect by the *daf-16b/FoxO* isoform^20^ suggests that neuronal DAF-16/FoxO might play an important role (activation or suppression) in conveying pheromone signals. To test whether the tissue specificity of DAF-16/FoxO is important in conveying pheromone signals, we performed rescue experiments using *ges-1* promoter-driven intestine-specific *daf-16/FoxO* and *unc-119* promoter-driven pan-neuronal *daf-16/FoxO*. Whereas intestine-specific DAF-16/FoxO did not rescue repulsion responses, neuron-specific DAF-16/FoxO recovered the repulsion responses of *daf-16/FoxO* mutants (Fig. 1b), indicating a tissue-specific (neuronal) expression of DAF-16/FoxO seems critical in conveying pheromone sensory signals in head neurons.

To identify neurotransmitters required for the transmission of pheromone sensory signals and to define the role of neuronal *daf-16/FoxO*, we tested several strains with mutations of genes involved in neurotransmitter signaling for their elicitation of repulsive behaviors. They are; *eat-4* (glutamate transporter), *tph-1* (serotonin biosynthesis), *cat-2* (dopamine biosynthesis), *egl-3* (pro-neuropeptide processing), *cha-1* (acetylcholine biosynthesis), and *unc-49* ﻿GABA ﻿receptor. Of these, only *eat-4*(*ky5*) mutants showed defective repulsion responses (Fig. 1c), suggesting that neuronal pheromone signals may share (or converted to) glutamate signals to elicit repulsion responses. When we repeated this experiment with another allele of *eat-4* (*ad819*) mutant, the results remained essentially the same (data not shown) (data in Fig. 1c).

We next assessed the genetic epistatic relationship between glutamate and *daf-2* IIS by comparing single *daf-2* or *eat-4* mutants with *daf-2*(*e1370*) *eat-4*(*ky5*) double mutants. Whereas *daf-2* mutants, in which DAF-16/FoxO is highly activated, showed strong repulsion responses similar to those of wild-type worms, *daf-2 eat-4* double mutants showed defective repulsion responses similar to those of *eat-4* single mutants (Fig. 1d), suggesting that *eat-4*- mediated glutamatergic signaling may occur downstream of *daf-2* activity. However, we cannot exclude the possibility that *daf-2* signaling could be in parallel with glutamatergic signaling by modulating other related metabolic pathways. Next, to test whether *daf-16/FoxO* functions in glutamatergic cell autonomously or not, we examined if *daf-16/FoxO* expression in *eat-4*-expressing neurons is enough for the recovery of repulsion behavior in *daf-16* mutants by generating transgenic worms in which *daf-16* is expressed under control of the *eat-4* promoter in *daf-2*(*e1370*);*daf-16*(*mgDf50*) mutants. Interestingly, *eat-4* promoter-driven *daf-16/FoxO* rescued the *daf-16/FoxO* mutant phenotype (Fig. 1e), providing evidence of a cell-autonomous function of DAF-16/FoxO in controlling the glutamatergic neurotransmission central to pheromone sensory signaling. In addition, when we examined whether DAF-16 influences *eat-4* expression, we found that the transcript levels of *eat-4* remained unchanged in *daf-16* mutants (Fig. S2d). We also found that the reduced *daf-16/FoxO* response in *daf-16*(*mgDf50*) single mutant was comparable to that of *daf-2; daf-16/FoxO* double mutants (Fig. S3). Taken together, our data suggests that *daf-2* signaling maybe genetically upstream of glutamatergic signaling not by regulating expression of *eat-4* expression level but presumably by altering another components in glutamate signaling pathway.

### Neuronal DAF-16/FoxO controls glutaminase gene expression

**Figure 2:**
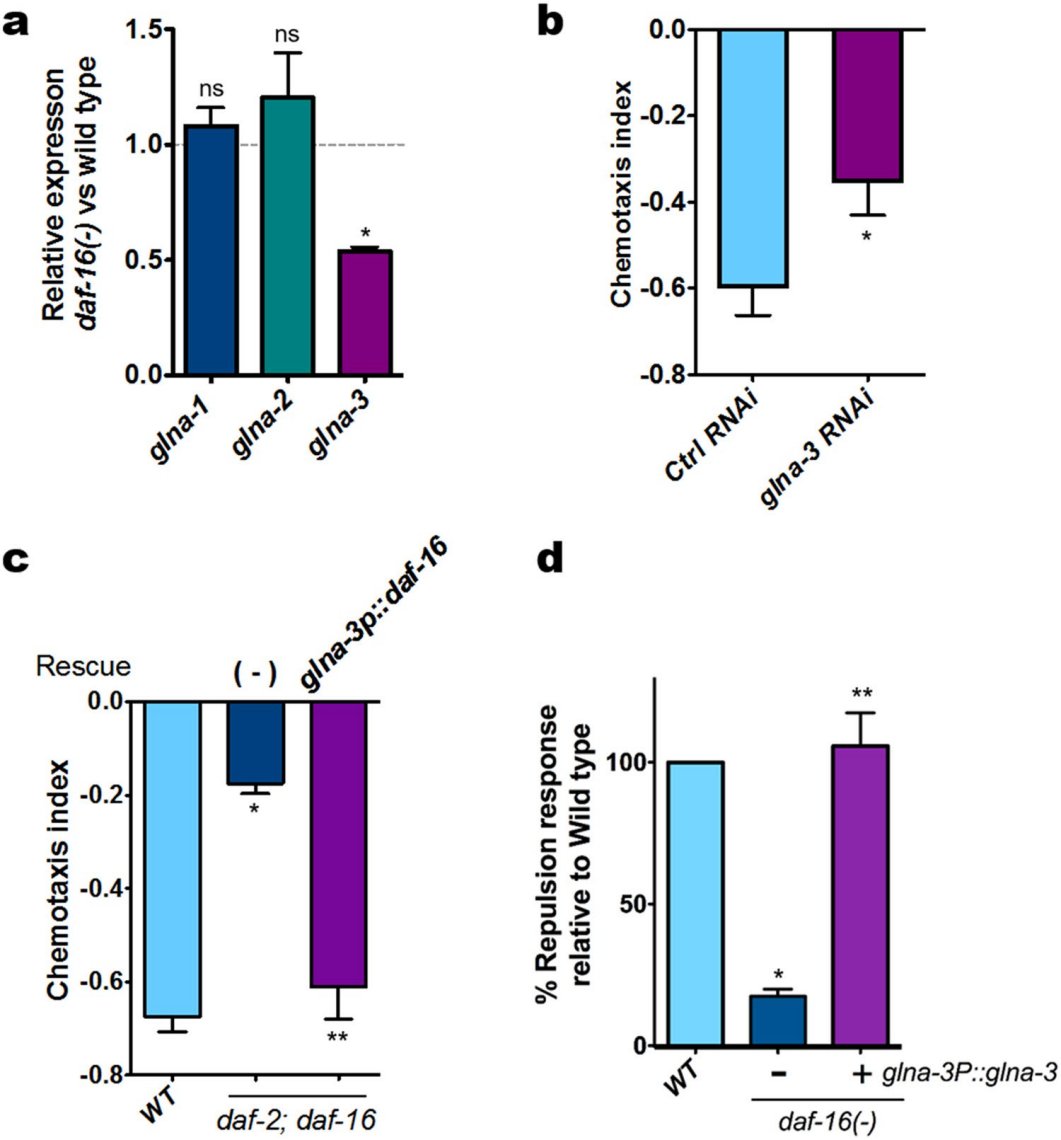
DAF-16/FoxO regulates *glna-3* expression. (**a**) Glutaminase gene transcript levels in *daf-16/FoxO* mutants. Bars represent the mean of three independent biological replicates. *P<0.05, ns: not significant compared to wild type. (**b**) RNAi against *glna-3* in a neuronal RNAi-sensitive strain (*unc-119P::sid-1*). F1 animals were hatched and grown on control or *glna-3* RNAi plates. F1 young adults were transferred to new RNAi plates and allowed to lay eggs, and F2 young adults were tested (Ctrl RNAi, *n* = 148; *glna-3* RNAi, *n* = 163). *P<0.05 compared to Ctrl RNAi (**c**) DAF-16/FoxO in *glna-3*-expressing neurons rescued the *daf-2;daf-16/FoxO* phenotype (wild type, *n* = 88; *daf-2;daf-16/FoxO, n* = 101; *daf-2;daf-16/FoxO*; *glna-3P::DAF-16/FoxO*, *n* = 82). *DAF-16/FoxO* cDNA was expressed under control of the *glna-3* promoter. *P<0.05 compared to wild type, **P<0.05 compared to daf-2;daf-16/FoxO. (**d**) *glna-3P::glna-3* rescued *daf-16/FoxO* mutant phenotype (wild type, n = 150; *daf-16/FoxO*, n = 149; *daf-16/FoxO;glna-3P::glna-3*, n = 150). *:*daf-16*(*-*) *vs daf-16*(*-*)*; glna-3P::daf-16*. Bars represent the mean of three independent biological replicates. Significance was determined using two-tailed, unpaired *t*-tests.

We next addressed a question as to what would be the potential role of neuronal DAF-16/FoxO in conveying the pheromone sensory signals through the glutamate neurotransmission to elicit repulsion behavior. The levels of neuronal glutamate are tightly regulated by glutaminase activity in conjunction with energy metabolism in astrocytes of mammalian brain^21^. In fact, mammalian brain is a high-energy demand organ and glucose is the primary source of energy. The *C. elegans* genome contains three glutaminase genes: *glna-1, glna-2*, and *glna-3*. To test whether DAF-16/FoxO modulates glutaminase gene expression thereby conveying pheromone sensory signals, we examined the relative expression of these genes and found that only *glna-3* expression was reduced in *daf-16/FoxO* mutants (Fig. 2a), which was also supported by RNAi knockdown results (Fig. 2b). We also found that the expression of DAF-16/FoxO in *glna-3*-expressing neurons rescued the repulsion responses of *daf-2* (*e1370*)*; daf-16/FoxO* (*mgDf50*) mutants (Fig. 2c). And the reduced response of *daf-16* mutant was fully rescued by overexpression of *glna-3*(*glna-3P::glna-3*), which suggests that *glna-3* acts downstream of *daf-16* to regulate the pheromone response (Fig. 2d, Fig. S7a). In this rescue experiment, independent transgenic lines were also tested and they all showed essentially the same results (Fig. S7a). Taken together, these results strengthen the notion that glutamatergic neuronal activity responsible for conveying pheromone sensory signals to elicit repulsion behavior appears to be regulated at the level of *glna-3* expression by neuronal DAF-16/FoxO. To corroborate the involvement of glutamate receptors in conveying pheromone sensory signals, we next tested worms with mutation of *mgl-1*, a homolog of the human type II metabotropic receptor GRM3, which is predicted to locate at the presynaptic glutamate neuron that inhibits glutamate release^22^. As expected, *mgl-1*(*tm1811*) mutants elicited a stronger repulsion response than wild-type worms (Fig. S4), perhaps due to enhanced presynaptic glutamate release, indicating the potential role of MGL-1 as a gate for pheromone-elicited glutamatergic repulsion responses. However, it remains to be determined whether additional downstream components of glutamate signaling (e.g., *glr-1*, *mgl-2*, and *nmr-2*) in post-synaptic neurons contribute to repulsion responses. Because the *mgl-1*(*tm1811*) strain exhibited a hypersensitive phenotype at a lower concentration of pheromone (i.e., >1.0 nM), we normalized the repulsion responses of *mgl-1* mutants to those of wild-type worms.

### Cellular and transcriptional expression of *glna-3*

**Figure 3:**
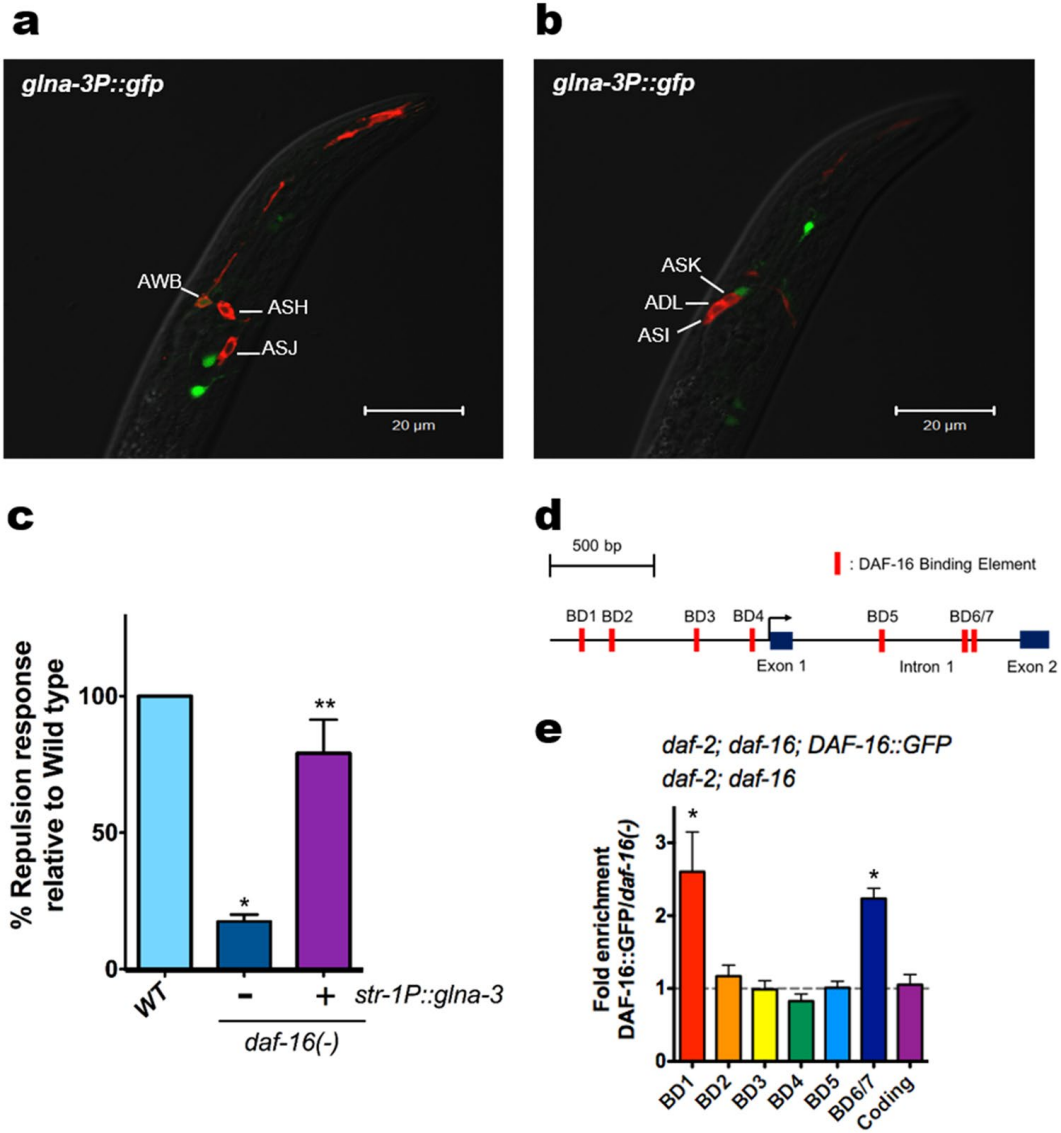
Expression pattern of *glna-3P:gfp* and ChIP analysis of DAF-16/FoxO::GFP bound to the upstream region of *glna-3 gene* (a) and (b) Expression pattern of *glna-3P::gfp*. Worms were also stained with DiI dye to visualize chemosensory amphid neurons. (c) *str-1P::glna-3* rescued *daf-16/FoxO* mutant phenotype (wild type, n=160; *daf-16/FoxO*, n=155; *str-1P::glna-3; daf-16/FoxO*, n=150.) *P<0.05 compared to wild type, **P<0.05 compared to *daf-16/FoxO*. (d) Putative DAF-16/FoxO binding sites in the 5′ upstream region and first intron of the *glna-3 gene*. (e) ChIP of DAF-16/FoxO::GFP with anti-GFP antibody in *daf-2;DAF-16/FoxO* and *daf-2;DAF-16/FoxO;DAF-16/FoxO::gfp* mutants. Bars represent the mean of three independent biological replicates. *P<0.05 compared to *daf-2;DAF-16/FoxO*. Significance was determined using two-tailed, unpaired *t*-tests.

With respect to *glna-3*-expressing neurons, we observed that *glna-3P::gfp* expression, driven by a 1422-bp segment in the 5′ upstream region of the *glna-3* gene, was localized in head neurons (Fig. 3a,b). Specifically, *glna-3P::gfp* expression and a dye filling assay, which stains chemosensory amphid neurons, showed that *glna-3* is expressed in AWB neurons (Fig. 3a), consistent with previous findings that *eat-4/vGlut1* is expressed in AWB neurons^23^. By contrast, ASI, ADL, ASK, ASH, and ASJ neurons did not express *glna-3P::gfp* (Fig. 3b).

However, the *glna-3P::gfp* reporter used in our study^24^ was expressed in a limited number of head neurons compared to that of previously reported^23^. This is presumably because our promoter construct did not include the first intron sequence. At least three pairs of amphid neurons (ASH, ADL, and AWB) are required for detecting either attractants or repellents^25^, with the AWB neuron being required for repulsion responses to 2-nonanone^26^. To address whether AWB neurons are involved in transmitting dauer pheromone-mediated hermaphrodite repulsion behavior, *glna-3* was specifically expressed in *daf-16* mutant under the control of AWB specific *str-1* promoter^26^ (Figs 3c, S7b﻿). The *str-1P::glna-3* partially rescued the reduced repulsion phenotype of *daf-16* mutant, suggesting that AWB neuron, at least in part, plays a role in pheromone-induced repulsion response. We further tested *lim-4*(*ky403*) and *ceh-37* (*ok272*) mutants. In *lim-4* and *che-37* mutants, neuronal cell fate of AWB neurons is altered, as a result, AWB neurons adopt AWC neuronal characteristics^27, 28^. Our study showed that the *lim-4* and *ceh-37* mutants conferred reduced repulsion behavior upon exogenous dauer pheromone (Fig. S5). Thus, it is likely that pheromone sensing signal that is initially perceived by GPA-3 is transmitted through AWB glutamatergic neuron where neuronal DAF-16/FoxO modulates glutaminase gene expression, resulting in elicitation of repulsion behavior. Of course, we cannot exclude the possibility that other neurons may also be involved in this process.

These results also raised additional questions: (1) What are the molecular mechanisms by which DAF-16/FoxO transcriptionally regulates *glna-3* expression? (2) Similar to nematode DAF-16 /FoxO, can the corresponding mammalian mFoxO3 regulate glutamate transmission in the hippocampus, a specific expression site of mFoxO3, a close homolog of *daf-16* ^29^. To answer the first question as to the transcriptional regulation mechanism by which DAF-16/FoxO controls *glna-3* expression, we examined seven predicted putative DAF-16/FoxO binding domains (BDs) located within the 5′ upstream and first intron region of the *glna-3* gene (Fig. 3d) for their binding to DAF-16/FoxO. To determine whether DAF-16/FoxO could bind to these sites, we performed chromatin immunoprecipitation (ChIP) in *daf-2*(*e1370*)*; daf-16*(*mgDf50*)*; daf-16/FoxO::gfp* animals using anti-GFP antibody. ChIP assay showed that DAF-16/FoxO binding is more enriched in BD1 (upstream) and BD6/7 (intron region) of the *glna-3* gene regulatory region, suggesting that neuronal DAF-16/FoxO may regulate *glna-3* transcription by binding to at least these two upstream regions of the *glna-3* gene in neurons (Fig. 3e). This result is also supported by a recent report that *glna-3* levels were up-regulated in *daf-2* mutants compared to *daf-2;daf-16/FoxO* double mutants^30^. Together, it is suggested that the DAF-16/FoxO transcription factor may modulate neuronal glutamate homeostasis by regulating glutaminase expression, which subsequently produces the repulsion behavior in response to the exogenous pheromones. However, it remains to further delineate the interactions between DAF-16/FoxO and the specific DNA sequences within the BD1 and BD6/7 of the *glna-3* gene.

### A conserved modulatory role of DAF-16/FoxO in glutamatergic neurotransmission

**Figure 4:**
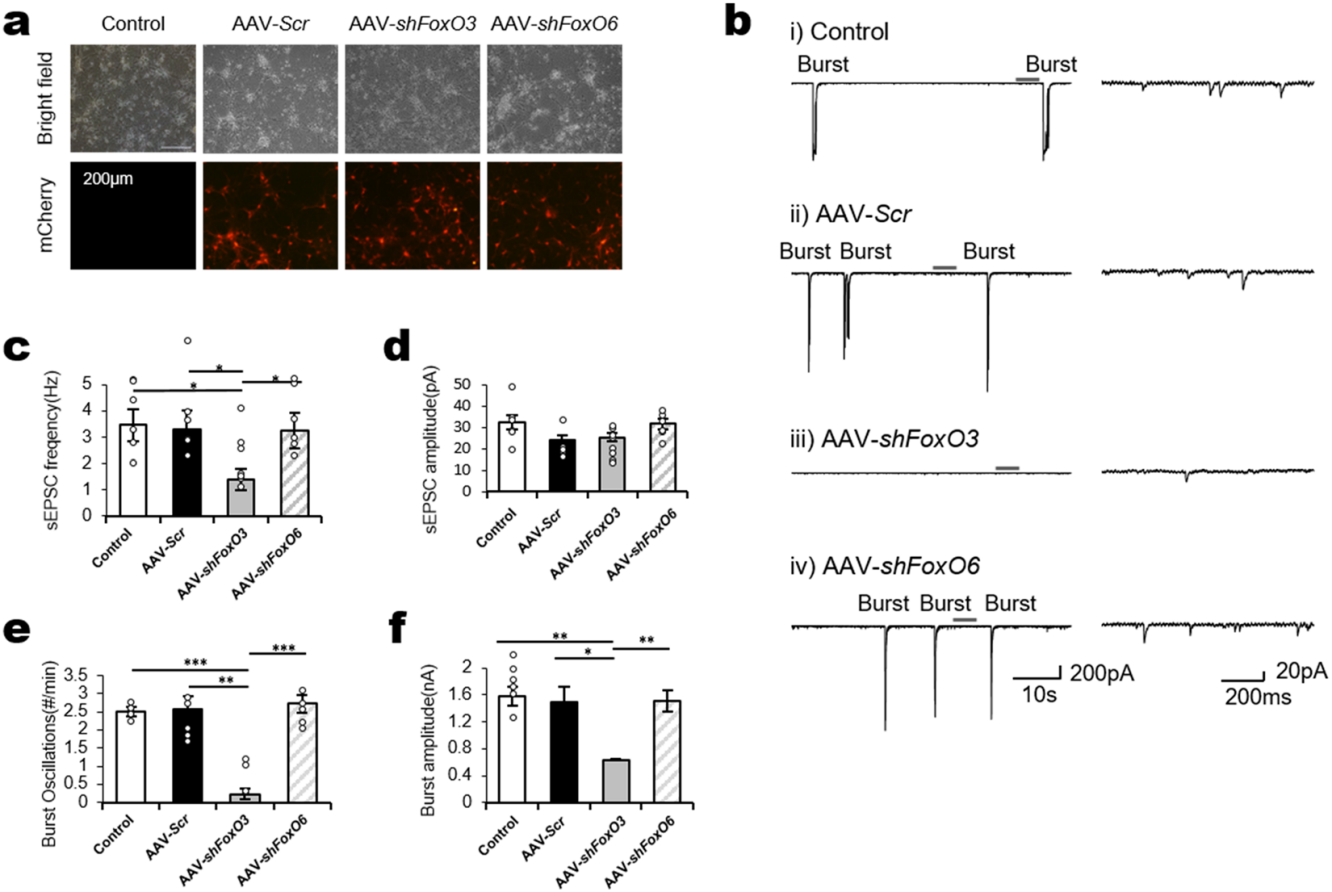
Knockdown of FoxO3 reduced sEPSCs, burst oscillations in mouse primary hippocampal neurons. (**a**) Primary hippocampal neurons without (control) or with infection of AAV-*Scr*, AAV-*shFoxO3*, or AAV-*shFoxO6* in bright-field (top) and mCherry fluorescence (bottom) images. AAV-infected groups had increased infection rates (38.1%, AAV-*Scr*; 42.5%, AAV-*shFoxO3*; 56.7% AAV-*shFoxO6*). (**b**) Representative traces of sEPSCs and burst oscillations from primary hippocampal neurons held at −70 mV in voltage clamping mode in the presence of picrotoxin. The part of each trace in the left panel marked with an upper line is enlarged in the right panel. (**c**) AAV-*shFoxO3* neurons (*n* = 8) exhibited fewer sEPSCs than control (*n* = 6), AAV-*Scr* (*n* = 6), or AAV-shFoxO6 (*n* = 6) neurons. (**d**) There were no differences in sEPSC amplitude. (**e**) and (**f**) Burst oscillation frequency and amplitude were reduced in AAV-*shFoxO3* neurons. *P<0.05, **p<0.01, ***p<0.001.

To answer the second question as to conservation of FoxO function between nematodes and mammals, we performed electrophysiological experiments in mice. Whereas *C. elegans* has only one FoxO transcription factor (DAF-16/FoxO), humans and mice have four FoxO transcription factors (FoxO1, 3, 4, and 6). DAF-16/FoxO shares the highest sequence homology with mammalian FoxO3^29^, whereas the expression pattern of FoxO6 is enriched in brain tissues^31^. To examine similarities between mammalian FoxO (mFoxO) and nematode DAF-16/FoxO in glutamatergic transmission regulatory function that is crucial for pheromone sensory transmission, we knocked down both mFoxO3 and mFoxO6 expression in cultures of mouse primary hippocampal neurons, which are known to express both mFoxO3 and mFoxO6^32^, by shRNA-mediated viral infection. Our experiment was also based on the earlier report that neurons in hippocampus mainly release glutamate and GABA^33^. After shRNA constructs were initially tested in NIH/3T3 cell lines before viral packaging, we chose two shRNA constructs for each gene (Fig. S6). Whole-cell patch recordings were obtained from primary hippocampal neurons 5 days after infection with scrambled adeno-associated virus (AAV-*Scr*), AAV-*shFoxO3*, or AAV-*shFoxO6* at 10 days *in vitro* ^33^. The efficiency of viral infection was confirmed by mCherry expression as a marker of AAV vector (Fig. 4a). Spontaneous excitatory postsynaptic currents (sEPSCs) were recorded in the presence of picrotoxin (50 μM) to exclude inhibitory postsynaptic currents (Fig. 4b). Both sEPSCs and large-amplitude burst oscillations were observed in primary hippocampal neurons, as previously reported^32^. The frequency of sEPSCs was reduced in AAV-*shFoxO3*-infected neurons compared with control, AAV-*Scr-*, or AAV-*shFoxO6*-infected neurons, whereas the frequency of sEPSCs was unchanged in AAV-*shFoxO6* infected neurons (Fig. 4c). There were no differences between groups in sEPSC amplitude (Fig. 4d). AAV-*shFoxO3*-infected neurons also showed reduced burst oscillation frequency (Fig. 4e) and amplitude (Fig. 4f **)**. These results indicate that the specific knockdown of mFoxO3 suppresses glutamatergic transmission in mammalian neurons, which is consistent with our results in *C. elegans*. Our findings demonstrate that FoxO plays a conserved pivotal role in maintaining glutamate homeostasis in the mouse hippocampus and the head of *C. elegans*.

## Conclusions and perspectives

**Figure 5:**
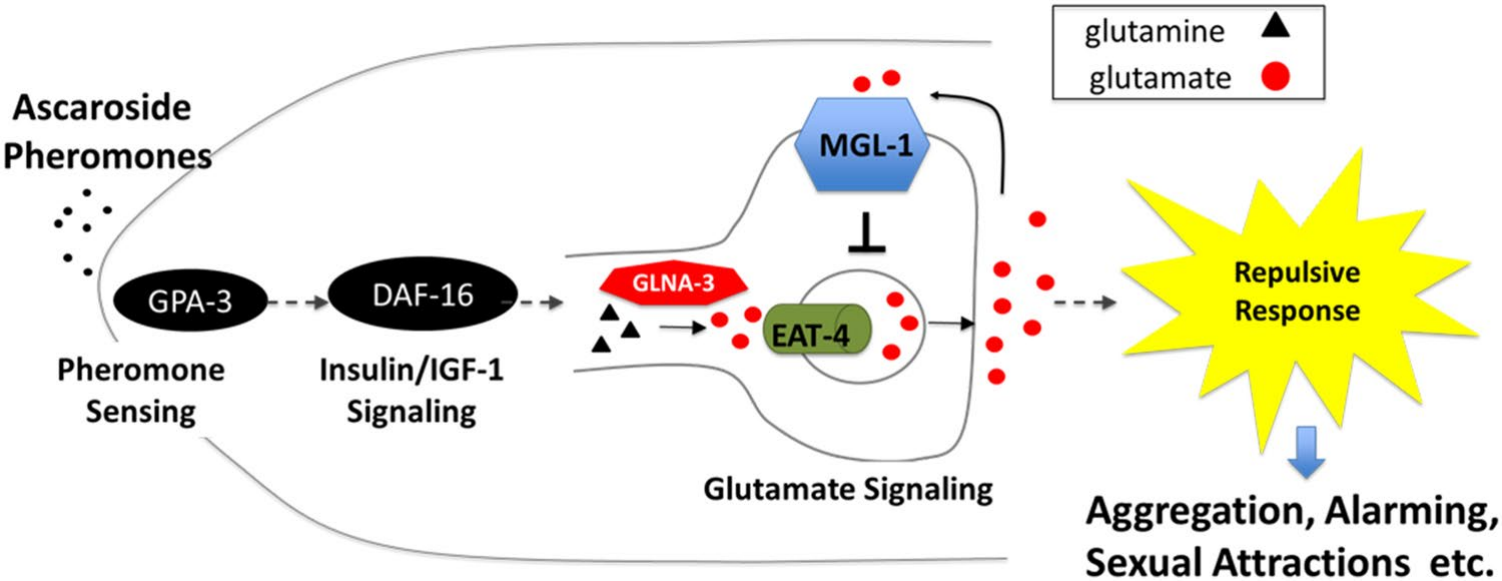
A proposed model of neuronal DAF-16/FoxO-mediated pheromone sensory signal transduction pathway. The pathway from pheromone perception to repulsion behavior includes at least five components: GPA-3, DAF-16/FoxO, GLNA-3, EAT4, and MGL-1. By binding to putative pheromone receptors (not shown), pheromones may stimulate GPA-3 and subsequently activate glutamatergic neurotransmission in AWB neurons, which is transcriptionally modulated by neuronal DAF-16/FoxO via GLNA-3 activation. The production of glutamate signals is likely gated by MGL-1/mGRM3.–unconfirmed relation; ─ confirmed relation.

In this work, we demonstrate how information contained in pheromones is processed internally by neural circuit to yield behavioral response. Furthermore, we provide a previously unexplored basic framework for neuronal components that are likely involved in neurotransmission of pheromone signals and a potential modulatory role of neuronal DAF-16/FoxO in this process. The potential components that participate in pheromone sensory processing leading to repulsion behavior include, but are not limited to, GPA-3, EAT-4, DAF-16/FoxO, GLNA-3, and MGL-1 (Fig. 5). Because *gpa-3* is not expressed in AWB neurons^17^, we may draw the conclusion that once pheromones are sensed in *gpa-3* expressing neurons such as ASI, ADL, or ASK, their signals are conveyed to *glna-3* expressing AWB neurons to elicit repulsion behaviors (Fig. 5). Moreover, this sensory process appears to be modulated by evolutionally conserved neuronal DAF-16/mFoxO3. Given that eliciting repulsion behaviors may be important for various pheromone activities^1, 2, 4^, our work on the identification of pheromone sensory signaling pathway may mark a major breakthrough in this field. ﻿This work could also stimulate investigations on general pheromone signaling in animals including mammals as well as its potential application to related neuronal disorders. As many important regulatory functions of FoxO across species are being explored, it may also be possible to conduct integrated studies that link neuronal FoxO-mediated pheromone sensation to neurological diseases caused by disturbances in glutamatergic neurotransmission in humans such as Alzheimer’s disease.

## Methods

### *C. elegans* strains and culture

*C. elegans* were cultured using standard techniques^34^. The strains used in this work were N2 Bristol (wild-type), *DAF-16/FoxO*(*mu86*), *DAF-16/FoxO*(*m26*), *DAF-16/FoxO*(*mgDf50*)*; daf-2*(*e1370*)*, DAF-16/FoxO*(*mgDf50*)*; daf-2*(*e1370*) *unc-119*(*ed3*)*; lpIs12[DAF-16/FoxOa::RFP* + *unc-119*(+)], *DAF-16/FoxO*(*mgDf50*)*; daf-2*(*e1370*) *unc-119*(*ed3*)*; lpIs13[DAF-16/FoxOb::CFP* + *unc-119*(+)]*, DAF-16/FoxO*(*mgDf50*)*; daf-2*(*e1370*) *unc-119*(*ed3*)*; lpIs14[DAF-16/FoxOf::GFP* + *unc-119*(+)]*, gpa-1*(*pk15*), *gpa-2*(*pk16*), *gpa-3*(*pk35*), *gpa-4*(*pk381*), *gpa-5*(*pk376*), *gpa-6*(*pk480*), *gpa-8*(*pk345*), *gpa-9*(*pk438*), *gpa-10*(*pk362*), *gpa-11*(*pk349*), *gpa-12*(*pk322*), *gpa-13*(*pk1270*), *gpa-14*(*pk342*), *gpa-15*(*pk477*), *goa-1*(*n1134*), *odr-3*(*n2105*), *eat-4*(*ad819*), *mgl-1*(*tm1811*)*, tbh-1*(*n3247*), *cmk-1*(*ok287*), *osm-6*(*p811*), *cat-2*(*e1112*), *egl-3*(*gk238*), *cha-1*(*n2411*)*, che-37*(*ok272*), *lim-4*(*ky403*) and *unc-49*(*e382*)*, DAF-16/FoxO*(*mu86*)*; ykpEx025[* *glna-3P::glna-3* + *myo-3P::rfp*], and *DAF-16/FoxO*(*mu86*); *ykpEx026[* *str-1P::glna-3* + *myo-3P::RFP*]. Worms were grown on nematode growth media seeded with *E. coli* OP50 as a food source.

### Transgenic worms

Rescue constructs of *DAF-16/FoxO* were generated by PCR fusion of the regulatory regions of *unc-119* (1200 bp), *eat-4* (2196 bp), or *glna-3* (1422 bp) upstream of the start codon of *DAF-16/FoxO::gfp* amplified from the TJ356 strain. Transgenes were microinjected in the germline of *daf-2; daf-16/FoxO* mutants with *myo-3P::dsRed* as a transgene marker. NC1478, a strain harboring wdEx584[*glna-3P::gfp, unc-119*(+)]; *unc-119*(*ed3*), was gift from Dr. David Miller III. Rescue construct of *glna-3* and AWB neuron-specific *glna-3* rescue construct were also generated by PCR fusion of the 1422 bp upstream regions of *glna-3* or 4000 bp upstream region of *str-1* gene^26^ to *glna-3* cDNA including 3′ UTR. Each transgenes were microinjected in the germline of *daf-16*(*mu86*) mutants with *Pmyo-3::RFP* as a transgene marker, to generate *glna-3* rescue worms and AWB neuron-specific *glna-3* rescue worms.

### Ascaroside Pheromones

All ascaroside pheromones (daumones 1–3 or ascr#1–3) were chemically synthesized and characterized at our laboratory as previously described^2, 5, 15^. Pheromones were dissolved in absolute ethanol and prepared in a stock solution (10 mM with ethanol). A serial dilution of pheromones were diluted into in M13 buffer in Eppendorf tubes to the final concentration of pheromone for the plate-based chemotaxis assay or drop assay (see below).

### Chemotaxis assay

For the plate-based chemotaxis assay, L1-synchronized worms were collected and grown to the young adult stage, washed three times with S-basal buffer to remove *E. coli*, and transferred to the center of a plate using aspirator tube assemblies for calibrated microcapillary pipettes (Sigma, St. Louis, MO). Chemotaxis index values were determined by counting worms that moved to different zones of the plate according to the following formula (see Fig. S1a): (A−B)/(A + B), where A is the number of worms that moved to zone A (containing pheromone [1 μM]) and B is the number of worms that moved to zone B (containing EtOH only). Unless otherwise indicated, chemotaxis index values were calculated 1 h after placement on the plate. In this assay, any anesthetizing drugs were not used. Worms that crawled up the side of the plate were not counted. To avoid errors in measurement due to reduced movement, we used strains with no motility deficits. Each data point represents 100–150 worms. Statistics were performed using GraphPad Prism 5. The drop assay for ascaroside pheromone-induced repulsion behavior was previously described^16^. For drop assay, worms were stage-synchronized with egg-preparation assay in prior to the assay. Twenty young adult animals (total 140–150 worms in each assay) were moved onto unseeded NGM plate (55 mm diameter) at 20 °C with the platinum wire. A serial dilution of pheromones (stock of 10 mM with ethanol) diluted into in M13 buffer in Eppendorf tubes to the final concentration of 1 μM of pheromone. Glass capillary was utilized to deliver pheromone to the head of a forward moving worm, then, scored the positive and negative responses. The repulsion behavior was monitored by putting a small drop of the ascaroside pheromone ahead of the forward moving worm and observed the two to three turns of backward movements as ‘repulsive’ and the fraction of worms ‘repulsive’ was calculated by comparing the with buffer controls. The synthetic daumone 1 used for the *mgl-1* mutant behavior was a different batch of other experiments.

### DiI staining and Microscopy

DiI staining was performed to visualize ciliated chemosensory neurons as described previously in Michael Koelle’s protocol (www.wormatlas.org/EMmethods/DiDiO.htm), with minor modifications. Briefly, DiI (1.1′-dilinoleyl-3,3,3′,3′-tetramethylindocarbocyanine perchlorate, Molecular Probes) stock solution was prepared in 2 mg/ml concentration in dimethyl formamide, stored at −20 °C. The DiI stock solution was diluted 1:200 in M9 and 150 μl of solution was put in a glass tube, where L2 worms were transferred and DiI-stained for 2 hours at 20 °C. After staining, worms were washed with M9 and transferred to NGM plate to crawl on a bacterial lawn for 1 hour to destain. Worms were visualized by using confocal microscope LSM 700 (Carl Zeiss). Images were analyzed with Carl Zeiss Zen 2.1 (Ver. 11.0) software.

### shRNA design and vector

pLKO.1-puro constructs containing scrambled (SHC002, Sigma), shFoxO3 (TRCN0000071616, Sigma), or shFoxO6 (TRCN000008777, Sigma) sequences were transfected into NIH/3T3 cells to confirm knock-down efficiency. The mouse shFoxO3 nucleotide targeted the FoxO3 sequence from 1441 to 1461 bp (5′-CGGCACCATGAATCTGAATGA-3′, NM_019740.2), and the mouse shFoxO6 nucleotide targeted the FoxO6 sequence from 830 to 850 bp (5′-CCTCGCCACTCATGTACCCAA-3′, NM_194060.1).

For AAV packaging, scrambled, shFoxO3, or shFoxO6 sequences were cloned into pAAV-U6-shRNA-CMV-mCherry vector by the site-directed mutagenesis method (Enzynomics). Each construct was synthesized using complementary primers; scrambled: 5′-AGAGATTGGTGCTCTTCATCTTGTTGTTTTTTCTCGAGTACTAGGA-3′ (sense), 5′-TGAATTGGTGCTCTTCATCTTGTTGAAACAAGGCTTTTCTCCAAG-3′ (antisense); shFoxO3: 5′-AGAGATCATTCAGATTCATGGTGCCGTTTTTTCTCGAGTACTAGGA-3′ (sense), 5′-TGAATCATTCAGATTCATGGTGCCGAAACAAGGCTTTTCTCCAAG-3′ (antisense); shFoxO6: 5′-AGAGATTGGGTACATGAGTGGCGAGGTTTTTTCTCGAGTACTAGGA-3′ (sense), 5′-TGAATTGGGTACATGAGTGGCGAGGAAACAAGGCTTTTCTCCAAG-3′ (antisense).

### Primary hippocampal neuron cultures

For primary hippocampal neuron cultures, hippocampi were isolated from mice on postnatal day 0–2 and maintained in ice-cold Ca^2+^- and Mg^2+^-free Hank’s balanced salt solution (HBSS). They were then incubated with HBSS containing trypsin (0.15 mg/ml) and L-cystein (0.5 mg/ml) for 20 min at 37 °C and triturated into single cells. After centrifugation, cells were suspended in Neurobasal A medium with B-27 supplement and 2 mM glutamine and then plated on coverslips coated with poly-D-lysine (1 mg/ml) at a concentration of 5 × 10^5^ cells/ml. Half of the medium was replaced every 4 days. Neuronal cultures were infected with AAV-*Scr*, AAV-*shFoxO3*, or AAV-*shFoxO6* at 10 days *in vitro* and used for experiments at 15 days *in vitro*.

### qRT-PCR analysis

Total RNA was isolated from age-synchronized young adult worms using Trizol reagent (Invitrogen) followed by clean-up with RNeasy spin columns (Qiagen, Valencia, CA). cDNA was synthesized using the Transcriptor First Strand cDNA Synthesis Kit (Roche) and used for qRT-PCR. All the relative expression data of worms by qRT-PCR was normalized by *act-2* gene expression.

### Electrophysiology

This experiment was performed as previously described^35^. Primary hippocampal neurons isolated from mice and cultured on coverslips were placed in a recording chamber (Warner Instrument, Hamden, CT) mounted to an upright microscope (EX51WI, Olympus, Japan) and camera (ORCA-R2, Hamamatsu, Japan). The recording chamber was perfused continually with artificial cerebrospinal fluid containing (in mM) 124 NaCl, 3 KCl, 1.3 MgSO_4_, 1.25 NaH_2_PO_4_, 26 NaHCO_3_, 2.4 CaCl_2_−2H_2_O, and 10 glucose aerated with 95% O_2_/5% CO_2_ at room temperature. Borosilicate glass capillaries (GC150F-10, Warner Instrument Corp., Hamden, CT) for fabricating patch electrodes (4 to 6 MΩ) were made using a pipet puller (P-97, Sutter Instrument, Novato, CA). Synaptic currents were measured in whole-cell configuration and amplified using Multiclamp 700B (Molecular Devices, Sunnyvale, CA). Data acquisition was performed using a Digitizer 1440 A (Molecular Devices) and Clampex 10.3 (Molecular Devices). Analysis of data was conducted using Clampfit 10.3 (Molecular Devices) and the MiniAnalysis program (Synaptosoft, Fort Lee, NJ). The intracellular pipette solution for voltage-clamp recordings contained (in mM) 130 CsCl, 10 MgCl2, 10 HEPES, 5 Mg-ATP, 5 QX-314, 0.5 Na-GTP, and 0.1 EGTA, at pH 7.3 and 282 mOsm. For measurement of bursting, membrane potential was held at −70 mV, and 50 μM picrotoxin (Sigma) was added to the bath for 5 min.

## Electronic supplementary material

Supplementary Information
