# Supplementary material for: A conserved neuronal DAF-16/FoxO plays an important role in conveying pheromone signals to elicit repulsion behavior in Caenorhabditis elegans
Source: Sci Rep. 2017 Aug 3;7:7260. doi: 10.1038/s41598-017-07313-6 (PMC5543152; doi:10.1038/s41598-017-07313-6)
Supplement: Supplementary file 1 — Supplementary Information [file 41598_2017_7313_MOESM1_ESM.pdf]

SREP-17-10421.R2 **Supplementary Information (clean version)**

Scientific Reports

A conserved neuronal DAF-16/FoxO plays an important role in conveying pheromone signals to elicit repulsion behavior in *Caenorhabditis elegans*

Donha Park[1]*, Jeong-Hoon Hahm[1,2]*†, Saeram Park[3], Go Eun Ha[4], Gyeong-Eon Chang[4], Haelim Jeong[1,2], Heekyeong Kim[2], Sunhee Kim[1,2]†, Eunji Cheong[4], and Young-Ki Paik[1,2,3]¶

[1]Department of Biochemistry, [2]Yonsei Proteome Research Center, [3]Department of Integrated Omics for Biomedical Science, [4]Department of Biotechnology, and College of Life Science and Biotechnology, Yonsei University, Seoul, Korea

*These authors contributed equally to this work.

†Present address: Center for Plant Aging Research, Institute for Basic Science (IBS), Daegu 42988, Republic of Korea

¶ Correspondence: Young-Ki Paik, Department of Biochemistry, College of Life Science and Biotechnology, Yonsei University, 50 Yonsei-ro, Sudaemoon-ku, 03722, Seoul, Korea

Email: paikyk@yonsei.ac.kr

Supporting Figures

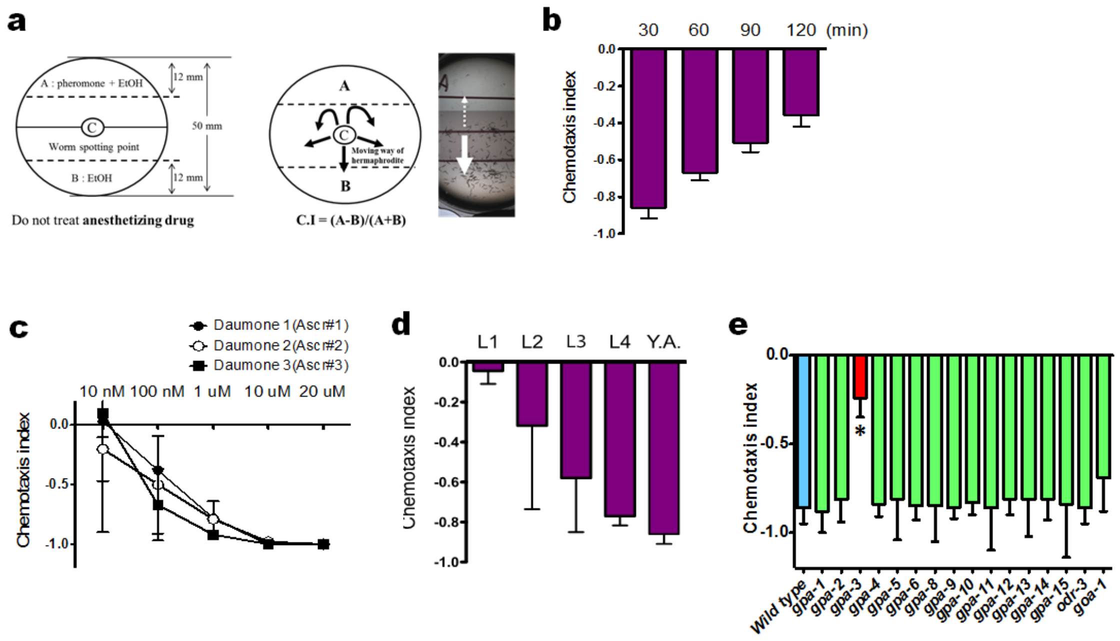

Fig. S1 Chemotaxis assay optimization and identification of *gpa-3* involved in ascaroside pheromone-induced repulsion responses.

a, Schematic depiction of plate based chemotaxis assay for *C. elegans* pheromone-induced repulsion responses. **b**, Time-dependent changes in repulsion response intensity in wild-type worms (1 uM of daumone 1 (ascr#1)). **c**, Concentration-dependent changes in repulsion responses in wild-type worms. **d**, Developmental stage-dependent changes in repulsion responses to the 1 uM of daumone 1 (ascr#1). Y.A., young adult. **e**, Of 17 various Gα subunit mutants, only *gpa-3(pk35)* mutant worms exhibited defects in pheromone-induced repulsion responses relative to wild-type worms in plate based chemotaxis assays (1 uM of daumone 1). *P<0.05

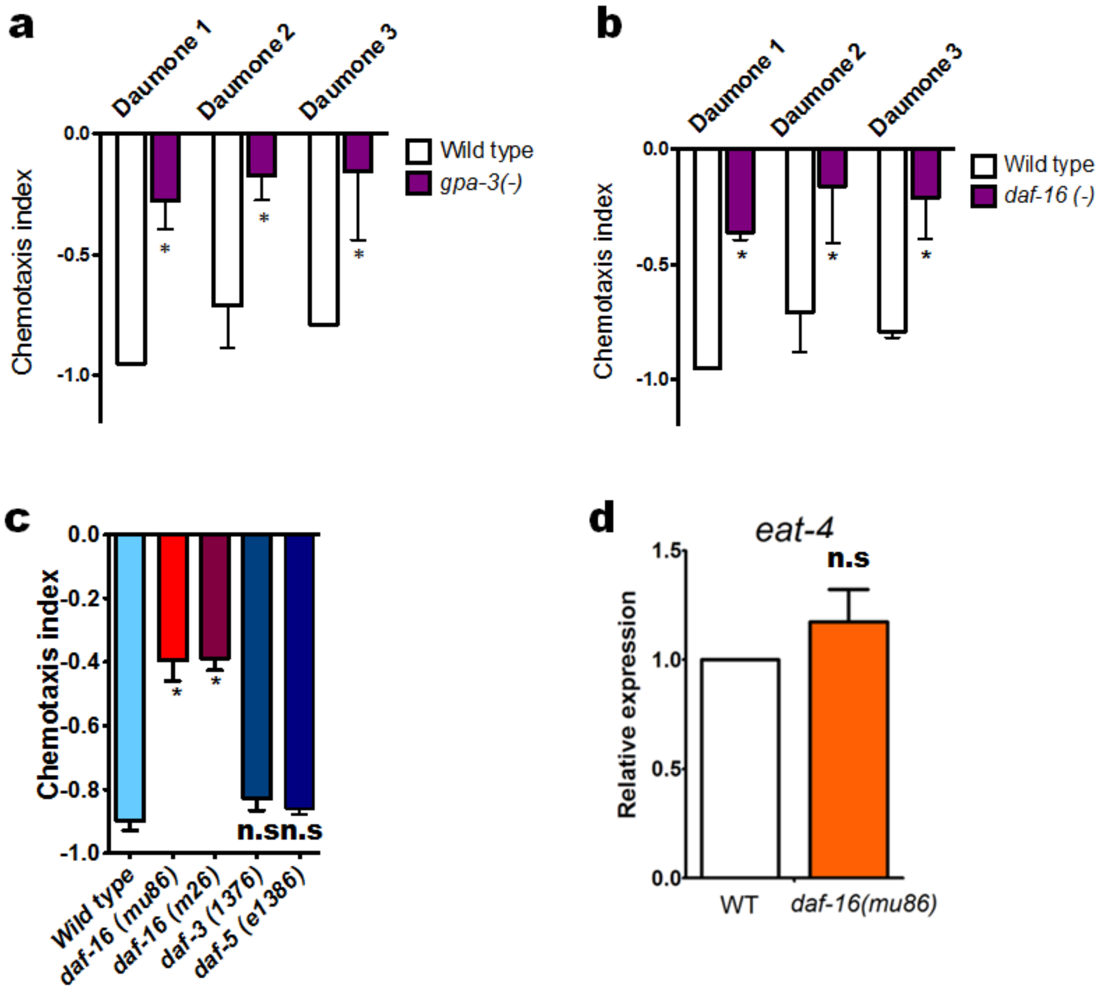

Fig. S2 Relative repulsion response and *eat-4* expression in the mutant strains

Ascaroside pheromone-induced repulsion responses of *gpa-3(pk35)* (**a**) and *daf-16(mu86)* (**b**) mutants. Shown here are each mutant's chemotaxis indices by all three major ascaroside pheromones (daumone 1-3, 1 uM). **c**, Defective repulsion responses in *daf-16*, *daf-3*, and *daf-5* mutants. The number of worms used was, WT, n=105; *daf-16(mu86)*, n=114; *daf-16(m26)*, n=129; *daf-3(e1376)*, n= 115; *daf-5(e1386)*, n= 122. Error bars represent standard error of the mean (SEM) in all figures. **d**, *eat-4* gene transcript levels in *daf-16(mu86)* mutants. Bar represents means of three independent biological replicates. **P*<0.05, n.s: not significant.

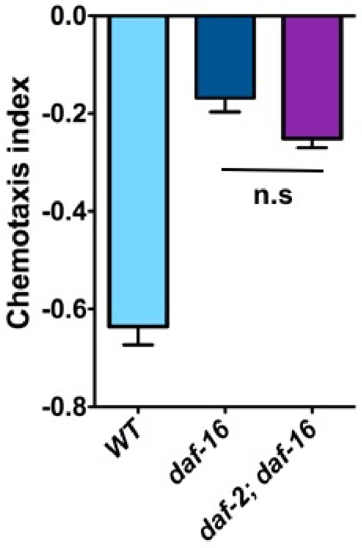

Fig. S3 Repulsion response in *daf-16* single and *daf-2; daf-16* double mutants

daf-16 single and *daf-2; daf-16* double mutant showed no significant differences in pheromone-induced repulsion response. (wild type, *n*= 170 ; *daf-16 n*= 185; *daf-2; daf-16* n=180). n.s, Not significant

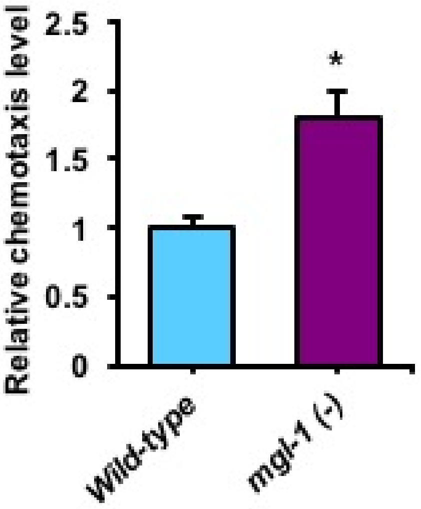

Fig. S4 Relative repulsion response in *mgl-1(tm1811)* mutant

Repulsion response of *mgl-1*mutants was assayed with lower concentration of pheromone1 (1 nM). *mgl-1* mutant showed stronger repulsion response than wild type worms upon 1 nM of pheromone (wild type, *n*= 270 ; *mgl-1*, *n*= 285). P < 0.05

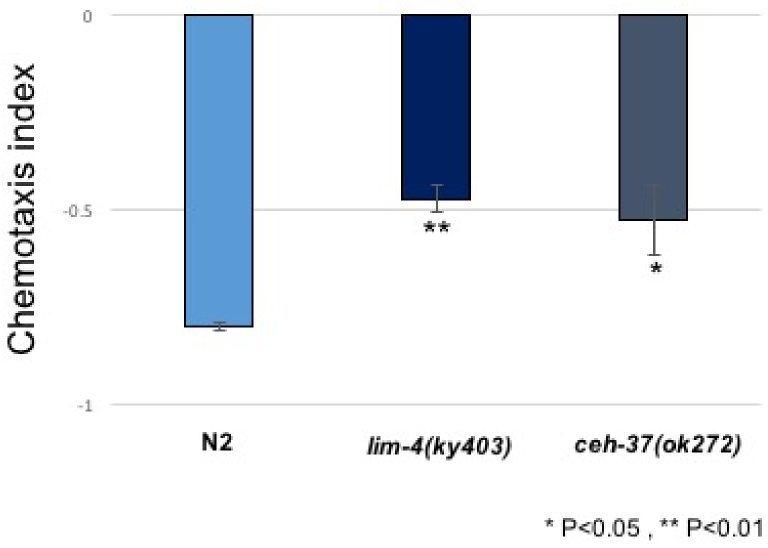

Fig. S5 AWB neurons may affect pheromone-induced repulsion behavior. *lim-4(ky403)* and *ceh-37(ok272)* mutants showed reduced repulsion behavior towards pheromone. Significance was determined using two-tailed unpaired *t*-tests. In these experiments, daumone 1 (1 uM) was used for three independent biological experiments. (Wild type, *n*=360; *lim-4(ky403)*, *n*=382; *ceh-37(ok272)*, *n*=364. Bars indicate means ± S.D.; *p < 0.05; **p < 0.01).

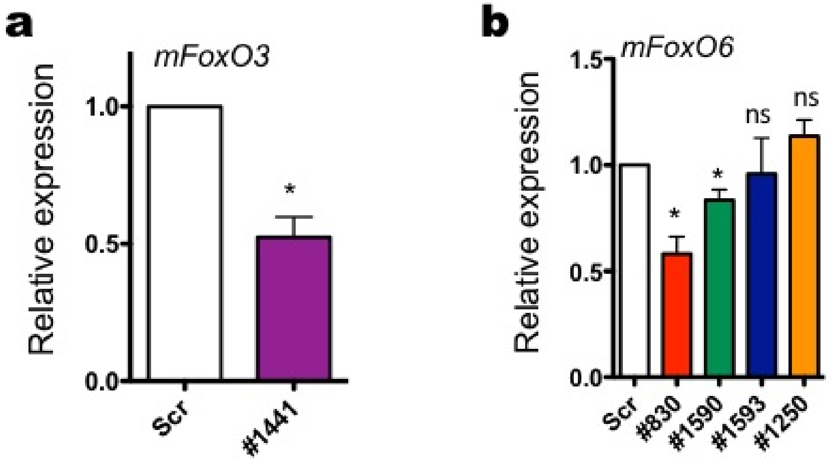

Fig. S6 mFoxO3 and mFoxO6 gene transcript. a, *mFoxO3* transcript levels after control transfection (Scr) or transfection with shRNA construct against the *mFoxO3* gene. **b**, *mFoxO6* transcript levels after control transfection (Scr) or transfection with shRNA constructs (#830, #1590, #1593, or #1250) against the *mFoxO6* gene. Bars are means of three independent biological replicates. *$P < 0.05$. n.s: not significant.

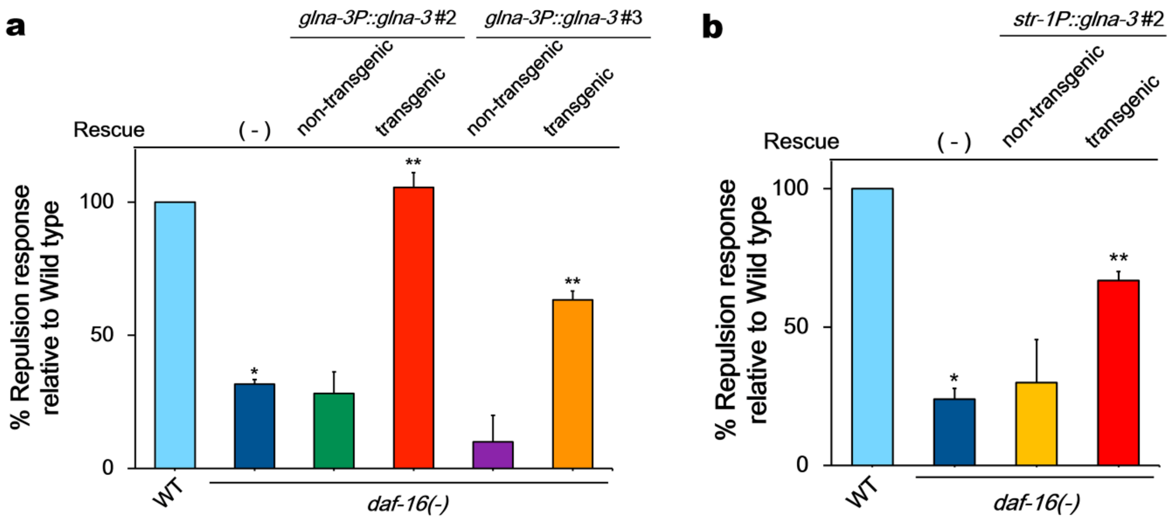

Fig. S7 *glna-3* expression rescues repulsion response of *daf-16/FOXO* mutant. a, *glna-3P::glna-3* rescued repulsion response of *daf-16/FoxO* mutant. Two independent transgenic lines (*daf-16/FoxO*; *glna-3P::glna-3* #2 and *daf-16/FoxO*; *glna-3P::glna-3* #3) with their non-transgenic siblings were examined. (wild type, n=50; *daf-16/FoxO*, n=50; *daf-16/FoxO*; *glna-3P::glna-3* #2 non-transgenic worms, n=48; *daf-16/FoxO*; *glna-3P::glna-3* #2 transgenic worms, n=50; *daf-16/FoxO*; *glna-3P::glna-3* #3 non-transgenic worms, n=50; *daf-16/FoxO*; *glna-3P::glna-3* #3 transgenic worms, n=50). *daf-16/FoxO*; *glna-3P::glna-3* #1 line rescuing result is shown in **Fig. 2d**. Non-transgenic siblings are worms without transgene marker that extrachromosomal transgenic moms laid. * P>0.05; *daf-16(-)* vs non-transgenic siblings, ** P=0.0113; non-transgenic siblings vs transgenic worms. **b**, *str-1P::glna-3* recued *daf-16/FoxO* mutant phenotype (wild type, n=65; *daf-16/FoxO*, n=65; *daf-16/FoxO*; *str-1P::glna-3* #2 non-transgenic worms, n=42; *daf-16/FoxO*; *str-1P::glna-3* #2 transgenic worms, n=60). *daf-16/FoxO*; *str-1P::glna-3* #1 line rescuing results are shown in **Fig. 3c**. * P>0.05; *daf-16(-)* vs. non-transgenic siblings, ** P=0.0218; non-transgenic siblings vs. transgenic worms. Bars represent the mean of two independent biological replicates. Significance was determined using two-tailed, unpaired *t*-tests.
